# COVID-19-related misinformation on social media: a systematic review

**DOI:** 10.2471/BLT.20.276782

**Published:** 2021-03-19

**Authors:** Elia Gabarron, Sunday Oluwafemi Oyeyemi, Rolf Wynn

**Affiliations:** a Norwegian Centre for E-health Research, University Hospital of North Norway, Sykehusveien 23, 9019 Tromsø, Norway.; b Department of Community Medicine, The Arctic University of Norway, Tromsø, Norway.; c Department of Clinical Medicine, The Arctic University of Norway, Tromsø, Norway.

## Abstract

**Objective:**

To review misinformation related to coronavirus disease 2019 (COVID-19) on social media during the first phase of the pandemic and to discuss ways of countering misinformation.

**Methods:**

We searched PubMed®, Scopus, Embase®, PsycInfo and Google Scholar databases on 5 May 2020 and 1 June 2020 for publications related to COVID-19 and social media which dealt with misinformation and which were primary empirical studies. We followed the preferred reporting items for systematic reviews and meta-analyses and the guidelines for using a measurement tool to assess systematic reviews. Evidence quality and the risk of bias of included studies were classified using the grading of recommendations assessment, development and evaluation approach. The review is registered in the international prospective register of systematic reviews (PROSPERO; CRD42020182154).

**Findings:**

We identified 22 studies for inclusion in the qualitative synthesis. The proportion of COVID-19 misinformation on social media ranged from 0.2% (413/212 846) to 28.8% (194/673) of posts. Of the 22 studies, 11 did not categorize the type of COVID-19-related misinformation, nine described specific misinformation myths and two reported sarcasm or humour related to COVID-19. Only four studies addressed the possible consequences of COVID-19-related misinformation: all reported that it led to fear or panic.

**Conclusion:**

Social media play an increasingly important role in spreading both accurate information and misinformation. The findings of this review may help health-care organizations prepare their responses to subsequent phases in the COVID–19 infodemic and to future infodemics in general.

## Résumé

**Objectif:**

Analyser la désinformation autour de la maladie à coronavirus 2019 (COVID-19) sur les réseaux sociaux durant la première phase de la pandémie, et discuter des moyens de lutter contre cette désinformation.

**Méthodes:**

Nous avons effectué une recherche dans les bases de données de PubMed®, Scopus, Embase®, PsycInfo et Google Scholar le 5 mai 2020 et le 1^er^ juin 2020 afin de trouver des publications liées à la COVID-19 et aux réseaux sociaux, plus précisément des études empiriques primaires consacrées à la désinformation. Nous avons suivi les éléments de rapport privilégiés dans le cadre des revues systématiques et méta-analyses, ainsi que l'outil de mesure permettant d'évaluer l'orientation des revues systématiques. La qualité des données probantes et le risque de biais dans les études retenues ont été classés en fonction de la catégorie d'estimation des recommandations, du développement et de l'approche adoptée en matière d'évaluation. La revue figure dans le registre prospectif international des revues systématiques (PROSPERO; CRD42020182154).

**Résultats:**

Nous avons identifié 22 études à inclure dans la synthèse qualitative. Le pourcentage de désinformation liée à la COVID-19 sur les réseaux sociaux représentait entre 0,2% (413/212 846) et 28,8% (194/673) des publications. Sur les 22 études examinées, 11 n'attribuaient aucune catégorie aux différents types de désinformation sur la COVID-19, neuf décrivaient des mythes spécifiques alimentant la désinformation, et deux signalaient les publications humoristiques ou sarcastiques en lien avec la COVID-19. Seulement quatre études se sont penchées sur les conséquences potentielles de la désinformation liée à la COVID-19: toutes ont indiqué qu'elle avait suscité de la peur ou de l'affolement.

**Conclusion:**

Les réseaux sociaux jouent un rôle de plus en plus important dans la propagation d'informations à la fois exactes et inexactes. Les résultats de cette revue pourraient aider les organismes de soins de santé à préparer leur réponse pour les prochaines phases d'infodémie sur la COVID-19, et pour les futures infodémies en général.

## Resumen

**Objetivo:**

Revisar la desinformación relacionada con la enfermedad por coronavirus 2019 (COVID-19) en las redes sociales durante la primera fase de la pandemia y debatir las formas de contrarrestar la desinformación.

**Métodos:**

Se realizaron búsquedas en las bases de datos PubMed®, Scopus, Embase®, PsycInfo y Google Scholar el 5 de mayo de 2020 y el 1 de junio de 2020 en busca de publicaciones relacionadas con la COVID-19 y las redes sociales que trataran sobre la desinformación y que fueran estudios empíricos primarios. Se siguieron los elementos de informe preferidos para revisiones sistemáticas y meta-análisis y las pautas para el uso de una herramienta de medición para evaluar revisiones sistemáticas. La calidad de la evidencia y el riesgo de sesgo de los estudios incluidos se clasificaron mediante el enfoque de valoración, desarrollo y evaluación de las recomendaciones. La revisión está registrada en el registro internacional prospectivo de revisiones sistemáticas (PROSPERO; CRD42020182154).

**Resultados:**

Se identificaron 22 estudios para su inclusión en la síntesis cualitativa. La proporción de desinformación sobre la COVID-19 en las redes sociales osciló entre el 0,2% (413/212 846) y el 28,8% (194/673) de las publicaciones. De los 22 estudios, 11 no categorizaron el tipo de desinformación relacionada con la COVID-19, nueve describieron mitos específicos de desinformación y dos informaron de sarcasmo o humor relacionado con la COVID-19. Solo cuatro estudios abordaron las posibles consecuencias de la desinformación relacionada con la COVID-19: todos informaron de que provocaba miedo o pánico.

**Conclusión:**

Las redes sociales desempeñan un papel cada vez más importante en la difusión tanto de información precisa como de desinformación. Las conclusiones de este estudio pueden ayudar a las organizaciones sanitarias a preparar sus respuestas a las siguientes fases de la infodemia de la COVID-19 y a las futuras infodemias en general.

## ملخص

الغرض مراجعة المعلومات الخاطئة عن مرض فيروس كورونا 2019 (كوفيد 19) على وسائل التواصل الاجتماعي خلال المرحلة الأولى من الوباء ومناقشة طرق مواجهة المعلومات الخاطئة.

الطريقة لقد بحثنا في قواعد بيانات PubMed®، وScopus، وEmbase®، وGoogle Scholar، في يومي 5 مايو/أيار عام 2020، و1 يونيو/حزيران عام 2020، عن المنشورات المتعلقة بكوفيد 19، ووسائل التواصل الاجتماعي التي تعاملت مع المعلومات الخاطئة، والتي كانت دراسات تجريبية أولية. قمنا باتباع العناصر المفضلة لإعداد التقارير للمراجعات المنهجية، والتحليلات التلوية، وأداة القياس، لتقييم إرشادات المراجعات المنهجية. تم تصنيف جودة الأدلة وخطر التحيز في الدراسات المشمولة، باستخدام تصنيف تقييم التوصيات، ونهج التطوير والتقييم. تم تسجيل المراجعة في السجل الدولي المستقبلي للمراجعات المنهجية (PROSPERO؛ CRD42020182154).

النتائج قمنا بتحديد 22 دراسة لإدراجها في السياق النوعي. تراوحت نسبة المعلومات الخاطئة عن كوفيد 19 على وسائل التواصل الاجتماعي من 0.2% (413/212846) إلى 28.8% (194/673) من المشاركات. من بين 22 دراسة، لم تقم 11 دراسة بتصنيف نوع المعلومات الخاطئة عن كوفيد 19، وقامت 9 دراسات بوصف معلومات مضللة خاطئة محددة، وأوضحت دراستان السخرية أو الدعابة المتعلقة بكوفيد 19. تناولت 4 دراسات فقط العواقب المحتملة للمعلومات الخاطئة عن كوفيد 19: وذكرت جميع الدراسات أن هذه المعلومات أدت إلى الخوف أو الذعر.

الاستنتاج تلعب وسائل التواصل الاجتماعي دورًا متزايد الأهمية في نشر كل من المعلومات الدقيقة والمعلومات الخاطئة. قد تساعد نتائج هذه المراجعة مؤسسات الرعاية الصحية في تجهيز استجاباتها للمراحل اللاحقة من تضليل المعلومات حول كوفيد 19، وغيرها من حالات التضليل المعلوماتي المستقبلية بشكل عام.

## 摘要

**目的:**

综述第一波新型冠状病毒肺炎疫情期间，社交媒体上相关的虚假信息，并探讨应对虚假信息的方式。

**方法:**

我们于 2020 年 5 月 5 日和 2020 年 6 月 1 日通过 PubMed®、Scopus、 Embase®、PsycInfo 及谷歌学术数据库，搜索了有关新型冠状病毒肺炎的发布信息以及涉及错误信息的社交媒体，其均为主要的实证研究。我们依据系统综述首选的报告项目，以及通过元分析和衡量工具，评估系统综述指导方针。利用推荐分级的评估、制定与评价方法，对纳入研究的证据质量和偏倚风险进行分类。该综述已在国际前瞻性系统综述注册网站注册 (PROSPERO; CRD42020182154)。

**结果:**

我们确定了 22 项研究纳入定性综合。社交媒体上有关新型冠状病毒肺炎的虚假信息占总发帖数的比例从 0.2％ (13/212 846) 到 28.8％ (94/673) 不等。22 项研究中，11 项没有对新型冠状病毒肺炎相关虚假信息进行分类，9 项描述了具体的虚假信息流言，两项报告了有关新型冠状病毒肺炎的讽刺或幽默。只有四项研究提出了新型冠状病毒肺炎相关虚假信息可能导致的后果：所有研究均报告了此情况会引发恐惧或恐慌。

**结论:**

社交媒体在传播信息（包括准确信息和虚假信息）方面越来越重要。总之，此次综述结果可能有助于医疗保健组织做好准备，应对新型冠状病毒肺炎信息疫情的后续阶段以及未来信息疫情。

## Резюме

**Цель:**

Составить обзор ложной информации, связанной с коронавирусным заболеванием 2019 г. (COVID-19), в социальных сетях во время первой фазы пандемии и обсудить пути противодействия распространению дезинформации.

**Методы:**

Авторы выполнили поиск публикаций, посвященных COVID-19 и социальным сетям, которые рассматривали ложную информацию и представляли собой первичные эмпирические исследования, в базах данных PubMed®, Scopus, Embase®, PsycInfo и Google Scholar по состоянию на 5 мая и 1 июня 2020 года. Авторы действовали в соответствии с предпочтительными компонентами процесса подготовки систематических обзоров и метаанализов и использовали инструменты измерения для оценки руководящих принципов по систематическим обзорам. Качество фактических данных и риск предвзятости включенных исследований классифицировались с использованием принципа оценки, разработки и изучения рекомендаций. Обзор зарегистрирован в международном проспективном реестре систематических обзоров (PROSPERO; CRD42020182154).

**Результаты:**

Было определено 22 исследования для включения в характеристическое обобщение данных. Доля ложной информации о COVID-19 в социальных сетях варьировалась от 0,2% (413/212 846) до 28,8% (194/673) опубликованных сообщений. 11 из 22 исследований не классифицировали тип ложной информации о COVID-19, 9 исследований описывали конкретные дезинформационные мифы, и 2 исследования рассматривали сарказм и юмор, связанные с COVID-19. Лишь 4 исследования рассматривали возможные последствия ложной информации о COVID-19, и все они сообщали о том, что такая информация сеяла страх и панику.

**Вывод:**

Социальные сети играют все более важную роль в распространении как точной, так и ложной информации. Результаты данного обзора могут помочь организациям здравоохранения подготовить меры реагирования для последующих фаз инфодемии COVID-19 и будущих инфодемий в целом.

## Introduction

The coronavirus disease 2019 (COVID-19) pandemic is spreading around the world with an increasing number of people becoming infected. Naturally the demand for information is high and people want to share news about the pandemic and their experiences. Social media have occupied a central role during the ongoing pandemic and the resulting wave of content related to COVID-19 has been referred to as an infodemic.^1^ However, incorrect information about COVID-19 can be dangerous because it may divert people away from taking appropriate actions that would help protect their health and the health of others and could lead them to take actions that may spread the illness or to engage in other problematic behaviours.^2^ The World Health Organization (WHO) has already recognized the importance of COVID-19-related misinformation and is participating in an awareness campaign aimed at encouraging people to check information with trusted sources.^3^

A distinction has been made between misinformation, defined as incorrect or false information that is shared without the intent to harm, and disinformation, defined as incorrect or false information that is shared with the aim of causing harm.^4^ However, making this distinction involves assessing the intent of the person spreading the information, which may be problematic.^5^^,^^6^ Consequently, in this review we use misinformation as a general term for incorrect or false information, regardless of intent. 

Our review focuses on misinformation that appeared early in the pandemic. During this phase, little was known about the virus, such as how it spread or how infected people could be treated most effectively. There was a shortage of protective equipment in many countries, no vaccines had been developed and it was uncertain how fast an effective vaccine could actually be produced. We believe this high degree of uncertainty during the initial phase may have been conducive to the appearance of a substantial amount of misinformation on social media. A synthesis of the evidence on COVID‐19-related misinformation on social media is needed to provide guidance for the health-care sector and to help in the assessment of guidelines for social media.

## Methods

We carried out a review of publications on COVID-19-related misinformation on social media that appeared during the first phase of the pandemic. The review followed guidelines detailed in the preferred reporting items for systematic reviews and meta-analyses and in a measurement tool to assess systematic reviews.^7^^,^^8^ The review is registered with the PROSPERO international prospective register of systematic reviews (CRD42020182154).

We searched the PubMed®, Scopus, Embase®, PsycInfo and Google Scholar databases on 5 May 2020 and 1 June 2020 for articles that included keywords related to social media and COVID-19. The full search strategy is detailed in Table 1 (available at: http://www.who.int/bulletin/volumes/99/6/20-276782). Articles were included in the review if they: (i) focused on COVID-19 and social media; (ii) considered misinformation; and (iii) were primary studies that reported findings. Articles that did not meet these criteria or were in a preprint version were excluded.

**Table 1:** Search terms, literature review of COVID-19-related misinformation on social media, 2020

Database	Search terms	No. of publications found	
		5 May 2020	1 Jun 2020
PubMed	social media OR social networking OR facebook OR twitter OR youtube OR whatsapp OR telegram OR instagram AND (2019 nCoV OR: 2019-nCoV OR: 2019nCoV OR: 2019 novel coronavirus OR COVID 19 OR COVID19 OR COVID-19 OR new coronavirus OR novel coronavirus OR SARS CoV-2 OR SARS-CoV-2 OR SARS CoV 2 OR SARS-CoV OR (Wuhan AND coronavirus))	73	138
Scopus	(ALL (“social media”) OR ALL (“social networking”) OR ALL (facebook) OR ALL (twitter) OR ALL (youtube) OR ALL (whatsapp) OR ALL (telegram) OR ALL (instagram) AND ALL (“2019 nCoV”) OR ALL (2019-ncov) OR ALL (2019ncov) OR ALL (“2019 novel coronavirus”) OR ALL (“COVID 19”) OR ALL (covid19) OR ALL (covid-19) OR ALL (“new coronavirus”) OR ALL (“novel coronavirus”) OR ALL (“SARS CoV-2”) OR ALL (sars-cov-2) OR ALL (“SARS CoV 2”) OR ALL (sars-cov) OR ALL ((wuhan AND coronavirus))) AND (LIMIT-TO (PUBSTAGE, “final”)) AND (LIMIT-TO (DOCTYPE, “ar”)) AND (LIMIT-TO (SRCTYPE, “j”))	31	387
Embase	(“social media” OR “social networking” OR facebook OR twitter OR youtube OR whatsapp OR telegram OR instagram) AND (“2019 nCoV” OR: 2019-ncov OR: 2019ncov OR “2019 novel coronavirus” OR “COVID 19” OR covid19 OR covid-19 OR “new coronavirus” OR “novel coronavirus” OR “SARS CoV-2” OR sars-cov-2 OR “SARS CoV 2” OR sars-cov OR (Wuhan AND coronavirus))	39	88
PsycInfo	(“social media” OR “social networking” OR facebook OR twitter OR youtube OR whatsapp OR telegram OR instagram) AND (“2019 nCoV” OR: 2019-ncov OR: 2019ncov OR “2019 novel coronavirus” OR “COVID 19” OR covid19 OR covid-19 OR “new coronavirus” OR “novel coronavirus” OR “SARS CoV-2” OR sars-cov-2 OR “SARS CoV 2” OR sars-cov OR (Wuhan AND coronavirus))	3	8
Google Scholar	“social media” OR “social networking” OR facebook OR twitter OR youtube OR whatsapp OR telegram OR instagram AND (“2019 nCoV” OR: 2019-ncov OR “novel coronavirus” OR “COVID 19” OR covid-19 OR “SARS CoV-2” OR sars-cov-2 OR sars-cov OR (Wuhan AND coronavirus))	217	367
**Total publications found**	NA	**363**	**988**

COVID-19: coronavirus disease 2019; NA: not applicable.

### Data analysis

References identified were uploaded to EndNote X9 (Clarivate Analytics, Philadelphia, United States of America) and duplicates were removed. Two authors examined the articles’ titles and abstracts, respectively, to assess their eligibility for inclusion in the study. In a second assessment round, the full texts of the articles selected in the first round were carefully analysed to confirm their eligibility by two independent reviewers. Doubts about eligibility were discussed with the third author until agreement was reached. Finally, the selected articles were divided among the three authors for data extraction and data were abstracted onto a specially standardized spreadsheet. The quality of the evidence in, and the risk of bias of, the articles were classified by two authors using the grading of recommendations assessment, development and evaluation (GRADE) approach.^9^

## Results

**Fig. 1:**
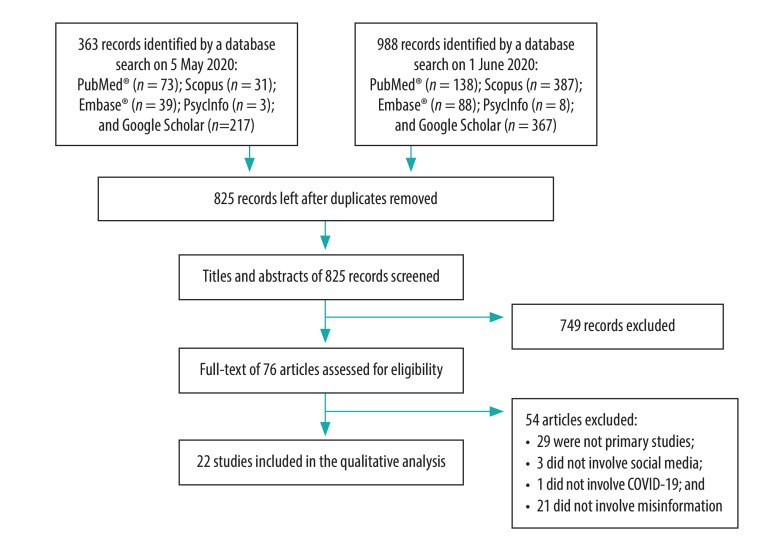
Study selection flowchart, literature review of COVID-19-related misinformation on social media, 2020

In total, we identified 1351 publications. After removing duplicates, we screened 825 titles and abstracts for eligibility and 22 articles finally met the inclusion criteria (Fig. 1).^2^^,^^5^^,^^10^^–^^29^ A list of articles whose full text was examined but which were excluded from the review is available in the data repository.^30^

COVID-19: coronavirus disease 2019.

Table 2 shows the main characteristics of the 22 studies included in the review; details of submission dates, funding and conflict of interests are available from the data repository.^30^ Fourteen of the 22 studies were cross-sectional and based on data extracted from social media, whereas eight were based on surveys or focus groups or both. Thirteen studies involved a single social media platform: Twitter (nine studies), Facebook (two studies), WhatsApp (one study) and YouTube (one study); the remaining nine studies involved several social media platforms. The monitoring period of social media ranged from 1 to 123 days. Two studies did not specify the monitoring period. According to GRADE evaluation criteria,^9^ 11 of the 22 studies were awarded 1 point, and the remaining 11 were awarded 2 points, which means they were all of low quality. However, the low quality was principally due to the studies being observational, whereas randomized trials, in contrast, provide the highest quality of evidence.^9^

**Table 2:** Study characteristics, literature review of COVID-19-related misinformation on social media, 2020

Study reference	Study design	Study period	GRADE score, points^a^	Type of social media studied	Social media or population sample	Type of misinformation reported	Reported effect of misinformation
Wahbeh et al.^28^	Cross-sectional	123 days (1 Dec 2019 to 1 Apr 2020)	2	Twitter	10 096 tweets	Misinformation in general	NR
Rufai and Bunce^24^	Cross-sectional	122 days (17 Nov 2019 to 17 Mar 2020)	2	Twitter	203 viral tweets from leaders of the G7 countries that had more than 500 likes^b^	Misinformation in general	NR
Kudchadkar and Carroll^16^	Cross-sectional	91 days (1 Feb to 1 May 2020)	2	Twitter	49 865 tweets	Misinformation in general	NR
Sharov^26^	Social media content analysis and survey	70 days (2 Mar to 10 May 2020)	2	Several, including VKontakte, Facebook, Instagram, Twitter and Odnoklassniki	3164 social media accounts and 903 survey respondents	Parallels drawn between COVID-19 and a possible Third World War, characterized by: (i) heaps of coffins; (ii) mass burials and overloaded crematoria; (iii) medical personnel wearing spaceman-like, anti-plague, protective medical clothing with gas masks; (iv) empty streets; and (v) closed or empty places of worship	(i) Fear; (ii) panic; (iii) misallocation of resources; (iv) stress experienced by health-care workers; and (v) “overheating of health-care sector”
Salaverría et al.^25^	Cross-sectional	31 days (14 Mar to 13 Apr 2020)	2	Several	292 hoaxes reported on certified platforms^c^	False health recommendations (e.g. alkaline diet or drinking wine), falsehoods related to health management, hoaxes falsely attributed to public health institutions and rumours about science and the origin of the coronavirus	NR
Subedi et al.^27^	Survey and focus group	27 days (27 Feb to 24 Mar 2020)	2	Facebook, Twitter, Instagram and YouTube	85 medical and dental interns	Misinformation in general	(i) Fear; and (ii) a man died and his wife was in a critical condition after they ingested chloroquine
Chesser et al.^12^	Survey	18 days (13–31 Mar 2020)	1	Several, including Facebook, Instagram, Twitter and Snapchat	1136 survey respondents	Misinformation in general	NR
Gebbia et al.^13^	Cross-sectional	15 days (8–22 Mar 2020)	1	WhatsApp	446 cancer patients	Misinformation in general	NR
Pérez-Dasilva et al.^21^	Cross-sectional	14 days (28 Feb to 12 Mar 2020)	2	Twitter	34 505 social media users and 37 362 of their interactions with other users	A media war between Republicans and Democrats in the United States that used COVID-19 as a story line	NR
Jimenez-Sotomayor et al.^5^	Cross-sectional	10 days (12–21 Mar 2020)	1	Twitter	351 tweets	Misinformation in general	NR
Kawchuk et al.^14^	Cross-sectional	8 days (24–31 Mar 2020)	2	Facebook	1350 social media users, including website users	Effect of chiropractic treatment on specific conditions, including pregnancy and immune responses (e.g. in COVID-19)	NR
Ahmed et al.^2^	Cross-sectional	8 days (27 Mar to 4 Apr 2020)	1	Twitter	2328 tweets	Conspiracy theory about the connection between 5G antennae and COVID-19	NR
Masip et al.^17^	Survey	8 days (3–10 Apr 2020)	2	Social media in general	1122 survey respondents	Misinformation in general	NR
Mustafa et al.^20^	Cross-sectional	7 days (13–19 Apr 2020)	1	Twitter	212 846 tweets	Sarcasm and humour related to COVID-19	NR
Mejia et al.^18^	Survey	6 days (15–20 Mar 2020)	2	Social media in general	4009 survey respondents	Social media exaggerates the severity of COVID-19	Fear of COVID-19 (16% of respondents strongly agreed and 25% agreed)
Morinha and Magalhaes^19^	Survey	6 days (23–28 Mar 2020)	1	Facebook	1198 survey respondents	Although the true origin of SARS-CoV-2 is still unknown, there were suggestions: (i) that it originated in bats or pangolins; (ii) that animal-to-human transmission occurred outside China; and (iii) that the virus emerged through laboratory manipulation	NR
Aker and Mıdık^11^	Survey	3 days (24–27 Mar 2020)	1	Several, including Facebook, Twitter, Instagram and WhatsApp	1375 medical students	Misinformation in general	NR
Pulido et al.^22^	Cross-sectional	2 days (6–7 Feb 2020)	1	Twitter	942 tweets	Suggestion that SARS-CoV-2 is a biological weapon and videos of people suddenly collapsing or having a seizure	NR
Pulido Rodríguez et al.^23^	Cross-sectional	2 days (6–7 Feb 2020)	1	Weibo and Twitter	1923 posts on Weibo and 1923 tweets	(i) Unproven treatments against COVID-19; (ii) pandemic as biochemical warfare; (iii) COVID-19 is a bioweapon or serves the interests of pharmaceutical companies; (iv) official information discredited; (v) false accounts of infection cases; and (vi) false information about the differences between COVID-19, flu and the common cold	NR
Yuksel and Cakmak^29^	Cross-sectional	1 day (1 May 2020)	1	YouTube	76 YouTube videos	Misinformation in general	NR
Ahmad and Murad^10^	Survey and social media content analysis	Not specified	2	Several, including Facebook, Instagram, Snapchat, YouTube and TikTok	516 social media users	Misinformation in general	Panic (26.6% of participants stated that, of all forms of information, false news about COVID-19 on social media created the greatest amount of panic)
Kouzy et al.^15^	Cross-sectional	Not specified	1	Twitter	673 tweets with more than five retweets	Humorous and non-serious comments	NR

COVID-19: coronavirus disease 2019; GRADE: grading of recommendations assessment, development and evaluation; NR: not reported; SARS-CoV-2: severe acute respiratory syndrome coronavirus 2.

^a^ Studies awarded 1 or 2 points using GRADE criteria are regarded as being of low quality; randomized trials provide the highest quality of evidence.

^b^ The 203 tweets were posted on the verified accounts of Shinzo Abe, Giuseppe Conte, Boris Johnson, Emmanuel Macron, Angela Merkel, Charles Michel, Justin Trudeau, Donald Trump and Ursula von der Leyen; the G7 countries are Canada, France, Germany, Italy, Japan, the United Kingdom of Great Britain and Northern Ireland and the United States of America.

^c^ The certified platforms were the Spanish accredited fact-checking platforms Maldita.es, Newtral and EFE Verifica.

### COVID-19 misinformation on social media

Six of the 22 studies reported the proportion of social media posts that contained misinformation on COVID-19, including false information and jokes. Four of these six studies reported the proportion on Twitter only, one reported the proportion on Twitter and Weibo and one reported the proportion on Facebook (Table 3). The proportion of misinformation ranged from 0.2% (413/212 846) to 28.8% (194/673) of posts.

**Table 3:** Proportion of social media posts about COVID-19 containing misinformation, literature review of COVID-19-related misinformation on social media, 2020

Study reference	Type of social media studied	Total no. of tweets or posts sampled	Tweets or posts containing misinformation or jokes or both, no. (%)	No. of retweets or repostings of the tweet or post containing misinformation
Mustafa et al.^20^	Twitter	212 846	413 (0.2)	NR
Kawchuk et al.^14^	Facebook	1 350	97 (7.2)	NR
Pulido Rodríguez et al.^23^	Twitter	1 923	168 (8.7)	2 338
Pulido et al.^22^	Twitter	942	100 (10.6)	59 955
Pulido Rodríguez et al.^23^	Weibo	1 923	206 (10.7)	232
Jimenez-Sotomayor et al.^5^	Twitter	351	50 (14.2)	NR
Kouzy et al.^15^	Twitter	673	194^a^ (28.8)	NR

COVID-19: coronavirus disease 2019; NR: not reported.

^a^ Of the 194 tweets, 153 were classified as misinformation and 41 were classified as humour.

Eleven studies did not categorize the specific type of COVID-19-related misinformation, nine described specific misinformation myths and two categorized the misinformation as sarcasm or humour related to COVID-19 (Table 2). Sarcasm and humour can draw on hyperbole or false claims to make a point but typically the intent is not to misinform. However, if the person receiving the message does not understand it is a joke or sarcasm, they may end up being misinformed.

Only four studies examined the effects of misinformation: all reported that it led to fear or panic (Table 2). One of the four mentioned that misallocation of resources and stress experienced by medical workers were also possible consequences of misinformation.^26^ Another study found that 46.8% (525/1122) of survey respondents were tired of COVID-19 being the main theme across all media.^17^

### Proposed solutions

Sixteen of the 22 studies proposed one or several ways of tackling COVID-19-related misinformation. The most popular measure, mentioned in eight studies, was promoting and disseminating trustworthy information.^11^^,^^12^^,^^15^^,^^16^^,^^22^^,^^26^^–^^28^ Seven studies suggested addressing, containing or debunking misinformation:^2^^,^^5^^,^^10^^,^^14^^,^^15^^,^^26^^,^^27^ misinformation could be replaced by facts and accurate information, or health authorities could debunk myths and help answer people’s queries. Four studies mentioned increasing the health literacy of social media users:^10^^,^^23^^,^^27^^,^^28^ they highlighted the need to educate social media users on how to determine what information is reliable and to encourage them to assume personal responsibility for not circulating false information. Three studies proposed that social media should be supervised by an authority or government:^10^^,^^19^^,^^26^ misinformation could be addressed by the government providing more comprehensive reports on the current epidemiological situation. Three studies suggested introducing policies or regulations for social media,^20^^,^^27^^,^^29^ and two mentioned the need for more research.^22^^,^^23^ Six studies did not suggest any solutions.^13^^,^^17^^,^^18^^,^^21^^,^^24^^,^^25^

## Discussion

Studies done during the first phase of the COVID-19 pandemic found that between 0.2% and 28.8% of social media posts about COVID-19 could be classified as misinformation. The large variability observed in the proportion may have been due to differences in social media samples, methods or the definition of misinformation. Studies on social media carried out during previous pandemics also reported a large variation in the proportion of posts identified as misinformation: 4.5% of posts on Twitter about H1N1 influenza,^31^ compared with 23.8% of content posted on YouTube about Zika virus disease,^32^ and 55.5% of posts on Twitter about Ebola virus disease.^33^

The studies identified several COVID-19-related myths that were spread through social media but provided no clear evidence of the effects of this misinformation. However, a few studies reported that misinformation led to fear and panic and to people becoming tired of hearing about COVID-19. There is evidence that misinformation can evoke negative emotions,^34^ which could, in turn, further contribute to its spread.^35^

Although misinformation is not a new phenomenon, today it can spread rapidly on social media and potentially reach more than half the world’s population. The studies in our review proposed six main ways of tackling COVID-19-related misinformation: (i) disseminating trustworthy information; (ii) addressing, containing or debunking misinformation; (iii) increasing social media users’ health literacy; (iv) officially supervising media in general; (v) introducing policies and regulations for social media; and (vi) increasing research on the topic. These suggestions have been included in published proposals for managing infodemics.^1^^,^^36^

Recently, WHO launched social media chatbots in Rakuten Viber and WhatsApp to provide accurate information about COVID-19.^37^^,^^38^ Several studies confirm that health professionals and public health authorities could assist by debunking misinformation and providing true information.^2^^,^^5^^,^^10^^,^^14^^,^^15^^,^^26^^,^^27^ Correspondingly, WHO has created a specific webpage for correcting misinformation about the disease.^39^ However, although messages that debunk misinformation on social media may have the desired effect, it has been observed that such messages can also contribute to the persistence of misinformation.^40^

As social media users can easily lose track of what information can be trusted, teaching users how to identify reliable information is important.^10^^,^^23^^,^^27^^,^^28^ One way of educating users about what information is trustworthy is to mark misleading posts as such.^41^ In addition, nudging (i.e. prompting or encouraging) people to think about the accuracy of a social media post has also been proposed.^42^ However, some form of self-regulatory behaviour may already exist on social media, whereby a collective intelligence acts to identify and stop misinformation by not forwarding it to others.^43^

Officially supervising media in general and introducing regulations for social media are sensitive topics because both measures can conflict with freedom of the press and the principle of free speech. Nevertheless, they may be considered during a pandemic. In fact, several large social media companies have introduced policies on controlling false or manipulated information.^44^^–^^47^

A good strategy for tackling COVID-19-related misinformation could employ several or all of these proposed measures along with any new approaches that might appear. In addition, as misinformation appears to spread faster on some social media than on others,^48^ platform-specific strategies could be developed. However, further research is needed.^22^^,^^23^ Investigations into the effectiveness of different approaches to countering misinformation will provide valuable knowledge that could help governments fight misinformation in future pandemics or health emergencies.

Evidence on the proportion of COVID-19-related misinformation on different social media platforms is insufficient. Moreover, little is known about the relative importance of the different reasons why people propagate misinformation. Accounts that are not verified by social media platforms as authentic seem to spread more misinformation than verified accounts.^15^^,^^25^ However, we do not know if social media users respond differently to these different types of account. Nor do we have much understanding about the impact of misinformation spread by bots.^49^

Studies that examine the longitudinal development of misinformation and the effect of that development is needed. Although information posted on social media may encourage specific behaviours, it is difficult to attribute people’s actions solely to social media postings because other factors may have an equal or even more important influence on determining when people decide to act. 

In addition, the most effective strategies for tackling COVID-19-related misinformation are currently not known. Although there are many ongoing attempts to correct misinformation, we were unable to identify any study that examined the effects of these attempts, such as whether they enabled people to be better informed or helped them feel safer.

The study has several limitations. Our review considered only peer-reviewed articles that were published during the first few months of the COVID-19 pandemic. We did not explore the grey literature and we excluded a considerable number of non-peer-reviewed preprints. We also excluded one article that met the inclusion criteria because the full text was not available and it was not possible to obtain a copy from the authors.^50^ The low quality of the articles included in the study is an important limitation. Moreover, there was a high risk of bias because data were collected over a short time period and because several studies used only part of the collected data in their analyses. We could not conduct a meta-analysis, because of the small number of studies that reported the effects of COVID-19-related misinformation and the nature of these studies. 

In conclusion, our review found that COVID-19-related misinformation on social media is an important issue, both in terms of the amount of misinformation in circulation and the consequences for people’s behaviour and health. Despite rapidly growing scientific interest in the topic of misinformation, few studies have examined the scope of the problem, including why misinformation is spread, its impact and how best to tackle it. The impact of COVID-19-related misinformation could be reduced by: (i) social media users, who should avoid spreading it; (ii) social media platforms, which should identify it, label it as misinformation or remove it; and (iii) public health authorities and health providers, who should increase their presence and COVID-19-related activities on social media. Our review investigated only the initial phase of the pandemic; future developments are likely to result in new types of misinformation.

As more countries experience additional surges in severe acute respiratory syndrome coronavirus 2 infection rates, social media will come to play an increasingly important role in disseminating accurate information. The knowledge acquired in our review of COVID-19-related misinformation may help health-care organizations prepare their responses to subsequent phases in the COVID-19 infodemic and to future infodemics in general.
